# Data Sharing in a Time of Pandemic

**DOI:** 10.1016/j.patter.2020.100086

**Published:** 2020-08-14

**Authors:** Sarah Callaghan

**Affiliations:** 1 Cell Press, 50 Hampshire St, Cambridge, MA, USA

## Abstract

The Research Data Alliance COVID-19 Working Group brought together over 440 volunteer data experts in order to address key issues with data and software sharing that need to be dealt with in order to be better able to inform the research response to a global pandemic. The resulting document gives thorough, well-structured, and clear guidance on what is needed, now and in the future, to maximize timely, quality data sharing and appropriate responses in health emergencies.

## Main Text

This Preview overviews and discusses the Research Data Alliance COVID-19 Working Group publication “Recommendations and Guidelines on data sharing,” published in June 2020.^1^

During a time of global pandemic, the importance of good quality data in decision-making has been thrown into high focus. Much has been written in the academic and popular press about how many COVID-19 tests have been administered and how the counts of infection rates vary from country to country. In order to make good decisions about how to deal with the current situation and plan for the future, high quality, reliable, and trusted data are needed.

The Research Data Alliance is a global network of more than 10,800 data professionals and domain experts from 145 countries that have come together as a bottom-up organization in order to build the social and technical infrastructure to enable open sharing and re-use of data. In March 2020, in response to growing concern about COVID-19, the RDA COVID-19 working group was formed. The working group grew rapidly, with over 440 members as of June 2020, spread out over different sub-groups, each focusing on different areas of interest. The COVID-19 working group also engaged with the work of a host of other RDA Working Groups, as well as external stakeholder organizations, including the Global Indigenous Data Alliance and the Research Software Alliance.

The resulting recommendations and guidelines on data sharing,^1^ published in final form on Jun 30, 2020, are a thorough and comprehensive overview of how to share data (and research software) from multiple disciplines to inform response to a pandemic, along with guidelines and recommendations on data sharing under the present COVID-19 circumstances. It is a long document (more than 140 pages) but is very thorough and well structured.

It is not necessary for the reader to read the entire document. Instead, the guidance is split up into four research areas (clinical, omics, epidemiology, social sciences) with an additional four cross-cutting themes (community participation, indigenous data, legal and ethical considerations, research software). This was done to allow users to quickly find the section relevant to them, while at the same time allowing the production of the guidelines to be done quickly and in parallel.

There is a clear distinction drawn between guidelines and recommendations in this document. A guideline gives detailed advice about the practice of research data and software sharing, in order to help researchers to follow best practices to maximize the efficiency of their work and to act as guidance and a roadmap for future emergencies. A recommendation provides higher level and more generic advice, helping policymakers and funders to maximize timely, quality data sharing, and appropriate responses in health emergencies.

As well as the four research areas and four cross-cutting themes, a selection of foundational elements are identified and discussed. These are common aspects that occur across different research areas and are in turn divided up into challenges and recommendations. None of these common aspects are particularly novel, but they are worth re-iterating in this context because they are of significant importance. The challenges are identified as:

• a critical need for data sharing, while acknowledging the trade-off between timeliness and precision, and
• the lack of harmonized standards and context, including the lack of pre-approved data sharing agreements.

The importance of research software is emphasized as part of the harmonized standards and context. Research software is often developed and maintained in an *ad hoc* fashion, hence access information for the software developed for analysis is not noted consistently in papers and, if the software is available, it is often placed in arbitrary locations with no guarantee of its persistence.

The foundational elements recommendations are simple to say (but difficult to implement) and include:

• the need for a coordinated, cross-jurisdictional effort to foster global open science,
• the importance of infrastructure investment to take advantage of economies of scale,
• the importance of ensuring that the research outputs produced are FAIR (findable, accessible, interoperable, and reusable) and timely,
• the need for data management planning in the early stages of any research enterprise,
• the necessity for metadata standards, not only for the originating community, but also to allow researchers outside the domain to find and use the data,
• the importance of documentation for putting data and research into context, as well as providing the information needed to ensure that the research can be verified and reproduced,
• the use of trustworthy data repositories to facilitate data sharing and increase the FAIRness of data, and
• the importance of rapid publication and sharing of research, while balancing the rapid dissemination of findings against the dissemination of reliable findings.

The significance of the ethical and privacy consideration about patient data are emphasized, acknowledging the balancing act that needs to be done to preserve individual rights, while at the same time addressing public health concerns and objectives. The key here is the statement that access to individual data and documentation should be as open as possible and as closed as necessary, to protect privacy and reduce the risk of data misuse.

Similarly, the document addresses the need for legal frameworks to ensure that emergency data-related legislations activated during a pandemic clearly outline the permissions and restrictions of the legislations with regards to data ownership, consent models, publication rights, and permissions around data sharing. These legislations may be supported by the use of technical solutions that ensure anonymization, encryption, privacy protection, and data de-identification, which may increase trust in data sharing.

Each of the four research areas and four cross-cutting themes follow the same structure for their specific section. They start with a section on focus and description, then scope, followed by policy recommendations (suitable for policymakers, funders, publishers, and infrastructure providers), and finally guidelines (suitable for researchers, data stewards, research software engineers, and public health officials). This common structure allows for readers to quickly and easily find the sections of interest and relevance to them.

References in the text are provided as hyperlinks to the document or website referenced, and a full and extensive reference list is provided at the end of the document, supported by a published Zotero library.^2^ Along with the reference list, the document provides an extended glossary of terms and acronyms to support the reader and an overview of useful additional resources.

One of the guiding principles of the RDA is openness, hence previous drafts of this document, all of which were opened for public community comment, are also made available on-line at (https://www.rd-alliance.org/group/rda-covid19-rda-covid19-omics-rda-covid19-epidemiology-rda-covid19-clinical-rda-covid19-0). All of the documentation, membership, events, posts, and discussion for each of the sub-groups can also be found online, linked from the landing page.^1^ The resulting document is licensed under CC0 1.0 Universal (CC0 1.0) Public Domain Dedication, which allows anyone to copy, modify, distribute, and perform the work, even for commercial purposes, all without asking permission. As with all CC licenses, correct attribution is required.

A document this size is daunting, but the executive summary is an excellent place to get an overview of the topics and the key issues. Table 1 in particular gives an excellent overview of the top challenges, guidelines for researchers, and recommendations for funders/policymakers for each of the sub-groups and cross-cutting themes.

This work was executed in an intense period over just over 6 weeks, with five iterations, all of which were opened for public community comment. It represents a massive and important body of work from which data professionals can learn, in order to improve our research infrastructures and practices to be better able to cope with global health crises.
